# P300 Latency with Memory Performance: A Promising Biomarker for Preclinical Stages of Alzheimer’s Disease

**DOI:** 10.3390/bios14120616

**Published:** 2024-12-15

**Authors:** Manal Mohamed, Nourelhuda Mohamed, Jae Gwan Kim

**Affiliations:** Biomedical Science and Engineering Department, Gwangju Institute of Science and Technology, Gwangju 61005, Republic of Korea; manalalnosh@gm.gist.ac.kr (M.M.); nonoalhodaali@gm.gist.ac.kr (N.M.)

**Keywords:** EPR, P300, neuropsychology, asymptomatic AD, prodromal AD, early screening

## Abstract

Detecting and tracking the preclinical stages of Alzheimer’s disease (AD) is now of particular interest due to the aging of the world’s population. AD is the most common cause of dementia, affecting the daily lives of those afflicted. Approaches in development can accelerate the evaluation of the preclinical stages of AD and facilitate early treatment and the prevention of symptom progression. Shifts in P300 amplitude and latency, together with neuropsychological assessments, could serve as biomarkers in the early screening of declines in cognitive abilities. In this study, we investigated the ability of the P300 indices evoked during a visual oddball task to differentiate pre-clinically diagnosed participants from normal healthy adults (HCs). Two preclinical stages, named asymptomatic AD (AAD) and prodromal AD (PAD), were included in this study, and a total of 79 subjects participated, including 35 HCs, 22 AAD patients, and 22 PAD patients. A mixed-design ANOVA test was performed to compare the P300 indices among groups during the processing of the target and non-target stimuli. Additionally, the correlation between these neurophysiological variables and the neuropsychological tests was evaluated. Our results revealed that neither the peak amplitude nor latency of P300 can distinguish AAD from HCs. Conversely, the peak latency of P300 can be used as a biomarker to differentiate PAD from AAD and HCs. The correlation results revealed a significant relationship between the peak latency of P300 and memory domain tasks, showing that less time-demanding neuropsychological assessments can be used. In summary, our findings showed that a combination of P300 latency and memory-requiring tasks can be used as an efficient biomarker to differentiate individuals with AAD from HCs.

## 1. Introduction

Dementia is a syndrome that is characterized by symptoms such as difficulties with memory, language, problem-solving, and other cognitive skills that affect the person’s ability to perform everyday activities [1]. Alzheimer’s disease (AD) is a neurodegenerative brain disease that is related to aging, and it is the most common cause of dementia, being present in nearly two-thirds of individuals with dementia [1,2]. According to Statistics Korea, the senior population (individuals aged 65 or older) is increasing, accounting for 17.5 percent of the overall population in 2022. Among those seniors, 10% have dementia [3].

In order to diagnose AD, physicians, with the help of neurologists, implement a variety of methods and tools; for example, they obtain the patient’s family history; conduct cognitive, physical, and neurologic examinations; and perform brain imaging to determine the patient’s levels of beta-amyloid, a hallmark of AD [4]. As there are no currently available cures or treatments for AD, it is important to detect this disease in its very early stages to enable its prevention and delay the progression of symptoms [5].

Many studies have investigated AD in its very early stage, referred to as asymptomatic AD (AAD), and its preclinical stage, referred to as mild cognitive impairment (MCI) or prodromal AD (PAD). The techniques used to investigate AD mainly include the implementation of brain neuroimaging using PET and fMRI modalities and/or neuropsychological tests [3,6]. Despite their ability to determine the stage of AD with high accuracy and sensitivity, the modalities used in neuroimaging are expensive and require hospital visits.

Because neuropsychological assessments have frequently been used to characterize dementia related to AD over the past 4 decades [7], early impairment in cognitive domains has been investigated using these assessments as a stand-alone approach [8]. In this approach, multiple cognitive domains such as attention, language, memory, and visuospatial and executive functions are assessed using a well-defined test, and the result of the test is generally scored to give the final evaluation. Some cognitive functions, such as attention, memory, and executive functions, have been found to be impaired in subjects with AAD and PAD compared to healthy controls (HCs) [5,6,9,10,11]. An accelerated decline in memory has been proven among AAD groups [12]. In addition, a substantial amount of evidence shows that patients exhibit a decline in their executive skills during the transition from AAD to PAD [13]. Attention tasks such as the Digit Span Test, language tasks such as the naming test, memory tasks such as the word listing test, and executive functioning tasks such as the go–no-go and Stroop tests are used to predict a patient’s subsequent progression to AD [7]. Despite this, these neuropsychological tests are ineffective in classifying the early stages of AD and can generate many false positives [14,15].

Electroencephalography (EEG) is a noninvasive, quantitative, objective, and easy-to-handle screening tool that is used to accurately diagnose the early stages of AD [3,16]. By extracting the event-related potentials (ERPs) embedded in the EEG signal during experiments involving cognitively demanding tasks, variations in neural responses can be used to differentiate these stages with higher discriminability power than the available methods [16,17].

ERP components can be acquired through different tasks including cognition, working memory, and verbal fluency tasks [18]. The visual oddball task is one of the paradigms most widely used to assess cognitive abilities related to attention and memory [19]. It is an ERPology experiment in which two different stimuli are presented to the subject in random order. While one stimulus is presented frequently (75–80%), the other appears rarely (25–20%), and the subject should respond to the rare stimuli - by pressing a button for example [20]. The P300 component is mainly evoked in this experiment.

P300 is a large positive component that peaks at approximately 300 ms post-stimulus when the subject cannot predict what the next stimulus will be [17]. This component has been abundantly studied, and its ability to differentiate the mild and severe stages of AD progression has been proven [21,22,23]. P300 indices, i.e., the amplitude and latency of the waveform, vary across the different stages of AD [24]. Although the variation across these indices is agreed upon in the literature with regard to the differentiation of HCs from AD patients [25,26,27,28], the changes in individuals with AAD compared to normal healthy adults and those with PAD have not yet been explored. Therefore, research must be conducted in order to fill this gap and enrich the literature.

Furthermore, results vary regarding the distinction of MCI (PAD) subjects from normal healthy controls. While many studies have observed smaller amplitudes in MCI patients compared to HCs [22,29,30], others have reported that the amplitude of P300 components does not change [31,32,33]. When referring to the P300 latency, although some studies have reported prolonged latency in the MCI group [22,34], other studies have found no differences between those with MCI and normal healthy adults [31,32,33]. The lower amplitude and late latency of P300 components may be caused by impairments in the allocation of attentional resources, which is fundamental for the processing of memory tasks in the MCI group [35]. Indeed, these inconsistencies have to be effectively addressed to enable early medical intervention to suppress the development of AD.

Many studies have used combined assessment techniques to differentiate between the early stages of AD with greater accuracy. For example, some studies have investigated the correlation between P300 indices and neuropsychological test results as a biomarker for MCI [23,36,37,38], while others have combined the neuropsychological test results with neuroimaging biomarkers [6,39,40] to investigate AAD. These combined approaches were implemented mainly due to the fact that the assessment of the early stages of AD (AAD, PAD) is difficult and using a multidimensional approach could be the safest strategy until a defined AD onset is known and a method to track its preclinical progression is found [6].

This study aims to find a biomarker for the early screening of AD. While many studies have investigated the impairment of cognitive abilities in MCI (PAD) and AD patients using the P300 indices, to our knowledge, no previous study has used either the P300 indices alone or its correlation with neuropsychological test results to screen for AAD. In short, we hypothesized that patients in the AAD and PAD stages would have a lower P300 amplitude and a delayed P300 response when processing target information. We also hypothesized that those individuals would have a longer P300 latency when comparing the processing of target and non-target stimuli. This is mainly due to difficulties and deficits in the attention, memory, and executive function cognitive domains.

## 2. Materials and Methods

### 2.1. Participants

To acquire the EEG data, the National Research Center for Dementia and Chonnam National University Hospital (Gwangju, Republic of Korea) recruited senior citizens living in Gwangju and adjacent cities. A series of medical examinations, including MMSE, PET, MRI, and patient interviews, were conducted to diagnose the different disease stages. The guidelines established by National Institute of Neurological and Communicative Disorders and Stroke (NINCDS)-Alzheimer’s Disease and Related Disorders Association (ADRDA) Work Group [8], the National Institute on Aging and Alzheimer’s Association (NIA-AA) [41], and the International Working Group (IWG) (Cummings et al., 2013) [42] were followed when determining the subject’s stage of disease. Subjects suffering from mental and/or behavioral disorders were excluded.

Three groups of subjects were included in this study: HCs: cognitively normal subjects; asymptomatic AD (AAD): subjects with amyloid positivity in PET; and prodromal AD (PAD): subjects with mild cognitive impairment. A total of 79 subjects participated in this study: HC group (n = 35, mean age = 74.8 ± 4.7 years), AAD group (n = 22, mean age = 76.5 ± 5 years), and PAD group (n = 22, mean age = 79.6 ± 4.8 years).

The recruited subjects were blind to our experimental protocol, and written informed consent was provided by the participants prior to the experiment. The experimental procedure was approved by the international review board at Gwangju Institute of Science and Technology. Table 1 shows a summary of the demographic and psychometric variables, alongside the statistical comparisons for the three groups. These comparisons were performed using a one-way ANOVA test.

### 2.2. Assessment

#### 2.2.1. Behavioral and Neuropsychological Tests

The neuropsychological screening was performed according to the Seoul Neuropsychological Screening Battery (SNSB), which is a comprehensive neuropsychological evaluation tool that has been used in Korea since its first standardization in 2003. This tool provides a score for each cognitive domain in order to compare patients’ cognitive domain abilities and identify changes in their cognitive domain functions over time. Its second edition, SNSB-II, consists mainly of three categories, including basic information, cognitive function tests, and other indexes; the overall assessment is generally performed within 1:45 to 2 h. The cognitive function tests conducted in this study consisted of a total of 18 subsets, divided into five domains; these included attention (Digit Span Test (DST), including DST-Forward and DST-Backward), language and related functions (Repetition, Korean-Boston Naming Test (K-BNT) and its short form (S-K-BNT), Praxis Ideomotor test, Calculation test, and Comprehension test), visuospatial functions (Copy of the Rey Complex Figure Test (Rey CFT)), memory (Seoul Verbal Learning Test (SVLT), including Immediate recall, Delayed recall, Recognition and Recognition discriminability index; and Rey Complex Figure Test (RCFT), including Immediate recall, Delayed recall, Recognition, and Recognition discriminability index), and frontal/executive functions (Contrasting program, Go-no-go, Controlled Oral Word Association Test (COWAT), including animal, supermarket, and phonemic tests; and Korean-Color Word Stroop Test (K-CWST), including Word reading, Color reading, and Interference score, Digit Symbol Coding (DSC), and Korean-Trail Making Test-Elderly’s version (K-TMT-E)). For a detailed description of these tests and their norms, see reference [43].

#### 2.2.2. Event-Related Potentials (ERPs)Experimental Design

##### Experimental Design

The EEG data-collection setup consists of two main parts: a subject part and an EEG-acquisition part. Participants sit comfortably in the subject part, which is separated from the other part by black curtains to avoid any surrounding disturbances. The classical visual oddball task was used in this study to assess the participants’ basic cognition abilities.

**Figure 1:**
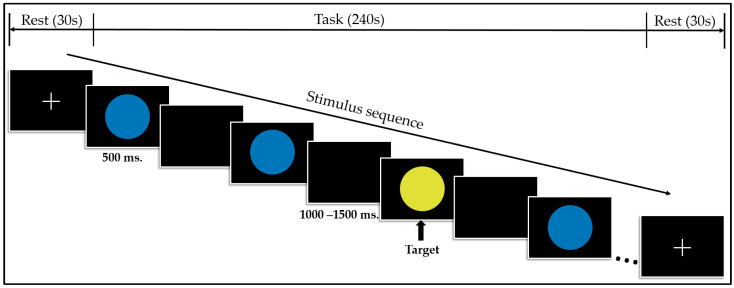
A schematic diagram of the sequence of the oddball task.

The participants were first asked to stare at the center of a computer monitor. A cross mark appeared for 30 s when the task first started. Then, a stimulus of blue and yellow circles appeared alternately in a random order, and the subjects were instructed to press a button when the yellow circle was presented. The ratio in which the two colors appeared was set to 75:25, so that the yellow circle appeared rarely compared to its blue counterpart; this served as the target stimulus. Each stimulus was presented for 500 ms, with a time gap of 1000 to 1500 ms between the stimuli (inter-stimulus interval). A total of 100 stimuli were presented during each data-acquisition process. Figure 1 shows a schematic diagram of the sequence and timing of the task stimulus.

##### Data Recording and Preprocessing

The EEG signal was recorded using a 32-channel dry-electrode wireless bio-signal acquisition system (g. Nautilus, g.tec, Taren Point, Austria) that was mounted to the subject’s head, according to the 10–20 international electrode positioning system. The left and right mastoids were used as referencing sites, and BCI2000 v3.6.R6143 software was utilized to record the signal. A band pass filter (BPF) with a half amplitude cutoff of 0.5 to 70 Hz and a 60 Hz notch filter were used to display the data. No referencing or filtering were used to record and store the data. The EEG signal was recorded with a sampling frequency of 500 Hz, alongside a fNIRS signal (beyond the scope of this study).

Offline data processing was applied to eliminate the power-line frequency, eye and muscle artifacts, as well as other artifacts. Both manual and automatic artifact rejection methods were applied.

##### Data Processing and Analysis

MATLAB EEGLAB/ERPLAB toolboxes were used to process and analyze the EEG data. First, raw EEG data were resampled to 250 Hz to reduce their size and improve the computational efficiency. Then, a second-order Butterworth IIR BPF with a half-amplitude cutoff of 0.5–30 Hz and a roll-off of 12 dB/oct was applied to eliminate unwanted noise. The 60 Hz power-line frequency was eliminated using a Zapline plug-in embedded within the ERPLAB. The P8 electrode site was used as the data reference to eliminate the common mode noise and allow a clearer view of the differential activity between electrodes.

Extended Infomax Independent Component Analysis (ICA) was implemented to remove the consistent blink (vertical) and saccadic (horizontal) eye movement artifacts interfering with the EEG data collected. Prior to performing the ICA, further processing steps were applied to improve the functioning of the ICA algorithm; these included the further filtering of the data using a BPF (1–30 Hz, roll-off = 48 dB/oct), the deletion of the time break intervals (30 s resting intervals before and after the task), and the deletion of unstable periods of the EEG, i.e., periods with deflection beyond that seen with ordinary artifacts. Then, the ICA was performed and the isolated components of eye blink, eye movement and muscle artifacts were removed from the data. The resulting ICA weights were then transferred to the original set obtained prior to the ICA preparation steps.

As a post-ICA processing step, bad channels were interpolated using the spherical interpolation method while the referencing channel was ignored. Then, the trials with target and non-target stimuli were segmented into 1000 ms epochs that were time-locked to the stimulus onset and a 200 ms pre-stimulus period was included for baseline correction.

In the final processing step, automatic as well as manual artifact detection procedures were applied to remove the reaming artifact from the data. In the automatic procedure, a simple voltage threshold, as well as moving window peak-to-peak algorithms with a threshold of ±100, and moving window width of 200 ms and step of 100 ms, were implemented. Meanwhile, during the manual rejection of artifacts, epochs with high levels of drift and noise that might affect the ERP waveform were excluded.

Finally, the remaining artifact-free epochs were averaged to obtain the P300 waveform, and the peak amplitude and peak latency were automatically measured between a 300 and 600 ms time window for all channels. The grand average waveforms from the C3, CZ, and C4 channels are shown in the results section.

**Figure 2:**
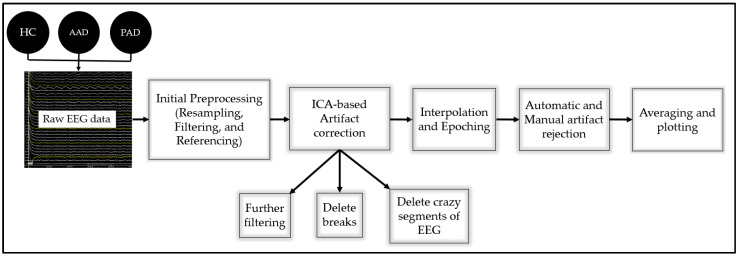
Flowchart of the EEG data processing steps.

A flowchart showing all of the processing steps is presented in Figure 2.

### 2.3. Statistical Analysis

Statistical analyses were performed using JASP 0.18.3.0. ANOVA tests were performed to determine the group differences using the P300 indices (latency and amplitude) and neurophysiological data.

To analyze the neuropsychological data, a one-way ANOVA test was performed, using groups (HC vs. AAD vs. PAD) as the independent variable and each neuropsychological test as the dependent variable. Further investigations were conducted using post-hoc analysis with Bonferroni correction applied to the ANOVA test results.

To analyze the ERP parameters, a mixed-design ANOVA test was first performed to investigate the effect of group-level differences between the processing of target and non-target stimuli at all channel sites. The sample was ranked according to conditions (target vs. not-target), channels locations (FP1, FP2, AF3, AF4, F7, F3, FZ, F4, F8, FC5, FC1, FC2, FC6, T7, C3, CZ, C4, T8, CP5, CP1, CP2, CP6, P7, P3, PZ, P4, PO7, PO3, PO4, PO8, OZ), and groups (HC vs. AAD vs. PAD). The conditions and channel locations were used as the within-subjects factor, while groups were used as the between-subjects factor. For a further comparison of the group differences, Bonferroni correction was applied to the ANOVA test.

To investigate which region of the brain exhibited the greatest difference among the three groups, a 2 × 4 × 3 × 3 mixed-design ANOVA test was performed; this used conditions (target vs. non-target), anterior–posterior distribution (frontal, central, parietal, occipital), and left–right distribution (left, middle, right) as the within-subjects factors and groups (HC vs. AAD vs. PAD) as the between-subjects factor.

Then, a 2 × 2 × 3 mixed-design ANOVA test was performed to check whether the central region channels (C3, CZ, C4) could be used as a sufficient biomarker to distinguish the difference between the groups. Conditions (target vs. non-target) and central channels (C3, CZ, C4) were used as the within-subjects factors while the groups (HC vs. AAD vs. PAD) were used as the between-subjects factor.

Finally, Pearson’s correlation coefficient (r) was assessed to find the link between the electrophysiological parameter (P300 latency) and the neuropsychological tests for HCs, AAD, PAD, and whole groups. To mitigate the risk of false positives, the False Discovery Rate (FDR) correction was applied. FDR-adjusted *p*-values served as the significance threshold for testing the associations between P300 latency and neuropsychological test scores.

## 3. Results

### 3.1. Neuropsychology

The psychological tests conducted in this study, including assessments of attention, language and related functions, visuospatial functions, memory, and frontal/executive functions, were evaluated across the HC, AAD, and PAD groups.

Repetition, SVLT (IR, DR, recognition, and recognition DI), RCFT (IR and DR), COWAT (/g/), K-CWST (CR and CR time per item), and ST interference differed significantly among the three groups (*p* = 0.007, 0.004, 0.042, 0.018, 0.018, 0.007, 0.023, 0.028, 0.001, 0.018 and 0.022, respectively). This results in a significant difference in the language and memory domains of the SNSB-II (*p* = 0.031 and 0.002, respectively), as well as in the SNSB-C (*p* = 0.028).

In the repetition test, post hoc analysis revealed no significant difference between the HC and AAD groups. However, the PAD group exhibited a significantly worse performance than the HC (mean difference = 0.227, *p* = 0.011) and AAD groups (mean difference = 0.227, *p* = 0.025). Although all other language and related function tests showed no significant differences in the post hoc pairwise comparison, a significant difference in the SNSB-II language domain was observed between the AAD and PAD groups (*p* = 0.038); in addition, a trending effect was observed between the HC and PAD groups (*p* = 0.103).

In the SVLT memory tests, post hoc analysis revealed no significant difference between the HC and AAD groups. However, significant differences were found between the HC and PAD groups for IR, DR, Recognition, and DI (*p* = 0.004, 0.046, 0.014, and 0.014, mean differences = 3.761, 1.691, 1.887, and 7.862, respectively), with the PAD group showing a worse performance. On the other hand, only IR differed significantly between the AAD and PAD groups, with *p* = 0.021 and a mean difference of 3.500.

Regarding the RCFT memory test, post hoc analysis revealed no significant difference between the HC and AAD groups. However, both RCFT IR and DR showed a significantly worse performance in the PAD groups compared to the HC (*p* = 0.028 and 0.044, respectively) and AAD groups (*p* = 0.011 and 0.044, respectively). The mean differences for these comparisons were 5.303, 4.648, 6.636, and 5.132, respectively. Table 2 summarizes the overall results of the neuropsychological test analysis.

Based on the results of the SVLT (IR, DR, recognition, and recognition DI) and RCFT (IR and DR), post hoc analysis indicates a highly significant difference in the SNSB-II memory domain between the HC and PAD groups (*p* = 0.004) and between the AAD and PAD groups (*p* = 0.011). Meanwhile, no significant difference was found between the HC and AAD groups.

For the frontal/executive function tests (COWAT:/g/ and K-CWST: CR), post hoc analysis revealed a significant difference between the PAD and AAD groups (*p* = 0.026 and 0.021, mean differences = 3.045 and 13.136, respectively). Furthermore, significant differences were noted in the K-CWST CR time per item, and in the ST interference between the HC and PAD groups (*p* = 0.001, 0.016, and 0.017, mean differences = 15.872, −0.238, and −0.188, respectively). However, the post hoc pairwise comparison for the SNSB-II frontal domain showed no significant differences.

### 3.2. ERP Results

Our hypothesis suggests that people in the early stages of AD, specifically AAD and PAD stages, may experience a lower P300 amplitude and a delayed P300 response when processing target information. We also expect that these individuals will have a longer P300 latency when focusing on target compared to non-target stimuli. This is likely due to difficulties with attention and increased demands on their working memory.

#### 3.2.1. Peak P300 Amplitude

The analysis of the peak P300 amplitude using ANOVA showed that in the within-subject effects, the comparison between conditions (target vs. non-target) was statistically significant (F (2,76) = 76.057, *p* < 0.001). However, the interaction between condition and group did not yield significant results (F (2,76) = 0.223, *p* = 0.801). Additionally, the group comparison for the between-subject effects did not reach statistical significance (F (2,76) = 0.583, *p* = 0.560).

**Figure 3:**
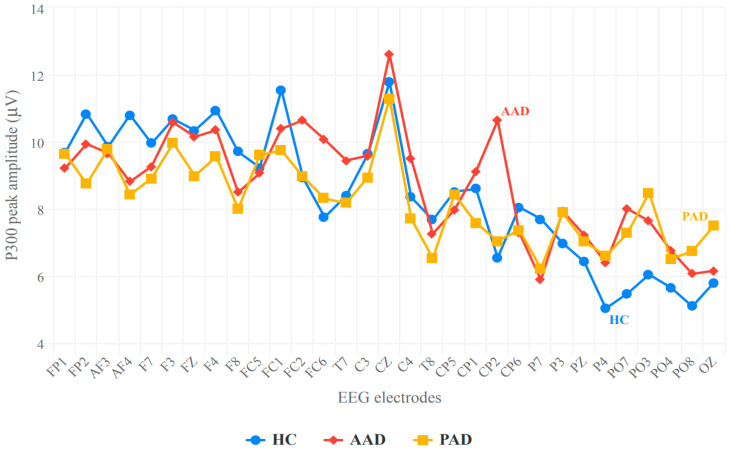
Mean peak amplitude values (µv) across the 32 channels during target processing in the HC, AAD, and PAD groups.

The averaged P300 peak amplitude values are presented in Figure 3. As shown, there is an overlap between these values for the HC and AAD groups, further indicating that there was no significant difference between them.

These findings lead to the conclusion that the peak P300 amplitude cannot serve as a biomarker for the differentiation of the three groups.

#### 3.2.2. Peak P300 Latency

When performing ANOVA for the peak P300 latency, the comparison between conditions (target vs. non-target) for the within-subject effects was statistically significant (*p* = 0.013). However, the interaction between conditions and group showed a trending effect (*p* = 0.111). In contrast, the group comparison for the between-subject effects reached statistical significance (*p* = 0.001).

Post hoc analysis revealed that the P300 peak latency during target processing did not differ significantly between the AAD and HC groups, with a mean difference of only −10.264 ± 12.784. However, a significant difference appeared when comparing the HC group to the PAD group, with a mean difference of approximately −53.607 ± 12.784 (*p* < 0.001). The P300 peak latency during target processing also differed significantly between the AAD and PAD groups, with a mean difference of −43.343 ± 14.167 (*p* = 0.041). These results indicate a slower response in the PAD group compared to the HC and AAD groups.

**Figure 4:**
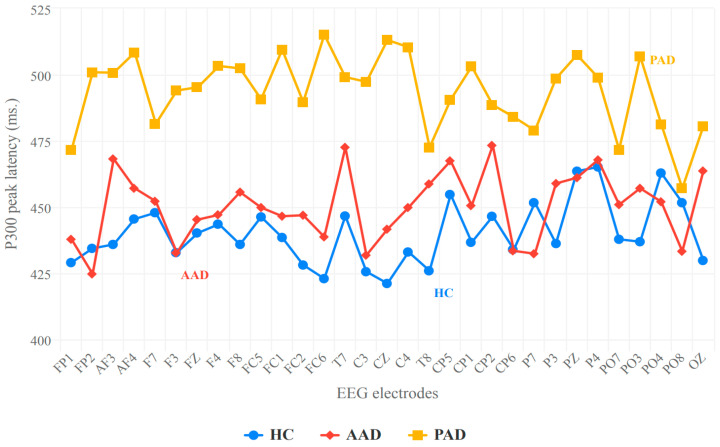
Mean peak latency values (ms) across the 32 channels during target processing in the HC, AAD, and PAD groups.

The averaged P300 peak latency values are summarized in Figure 4. As shown, there is a noticeable difference in the averaged peak latency between the HC and PAD groups across the 32 channels. However, the values for the HC and AAD groups overlap, showing no significant difference between them.

Hence, we conclude that the P300 peak latency can serve as a biomarker for the differentiation between those with AAD and PAD, as well as between HCs and those with PAD; however, it cannot effectively distinguish between those with AAD and HCs.

##### Optimal Regions for Distinguishing Groups Through P300 Latency

The ANOVA test conducted across the frontal (F3, FZ, F4), central (C3, CZ, C4), parietal (P3, PZ, P4), and occipital regions (PO7, OZ, PO8) of the scalp revealed a statistical significance (*p* = 0.002) between conditions (target vs. non-target) for the within-subject effects. Additionally, the group comparison in the between-subject effects reached statistical significance (*p* < 0.001).

Post hoc analysis revealed no significant difference in the P300 peak latency during target processing between the AAD and HC groups, with a mean difference of −8.619 ± 12.929. However, a significant difference was found when comparing the PAD group to the HC and AAD groups (mean difference = −53.907 and −45.288, *p* < 0.001 and = 0.030, respectively).

Among the four regions, the P300 peak latency in the central region was significant between the AAD and PAD groups during target processing (*p* = 0.014); meanwhile, no significant difference was observed between the AAD and HC groups. Meanwhile, the P300 peak latency in the central and parietal regions differed significantly between the HC and PAD groups during target processing (*p* < 0.001 and *p* = 0.013, respectively). In contrast, no significant difference was observed in the frontal and occipital regions among groups. These findings suggest that focusing on the peak P300 latency in the central region offers an effective means of distinguishing between groups.

##### Peak P300 Latency in the Central Region: Key to Group Distinction

**Figure 5:**
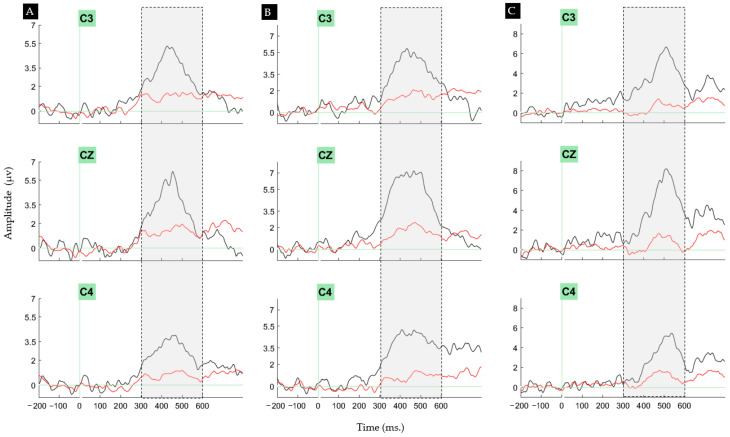
Average P300 waveforms recorded at the C3, CZ, and C4 electrodes during the presentation of target stimuli (shown in black) and non-target stimuli (shown in red) for the (**A**) HC, (**B**) AAD, and (**C**) PAD groups. The measuring window spans from 300 to 600 ms (shown in gray shadows).

Regarding the within-subject effects considered in the ANOVA test of P300 peak latency at C3, CZ, and C4, the comparison between conditions (target vs. non-target) was statistically significant (*p* < 0.001). The group comparisons in the between-subject effects also reached statistical significance (*p* < 0.001). Furthermore, the post hoc analysis indicated significant differences between the AAD and PAD groups during target processing, as well as between the HC and PAD groups (*p* < 0.001 for both comparisons). The grand average P300 responses at the C3, CZ, and C4 electrodes among the three groups are illustrated in Figure 5.

### 3.3. Correlation Between Neuropsychological Tests and P300 Peak Latency

**Figure 6:**
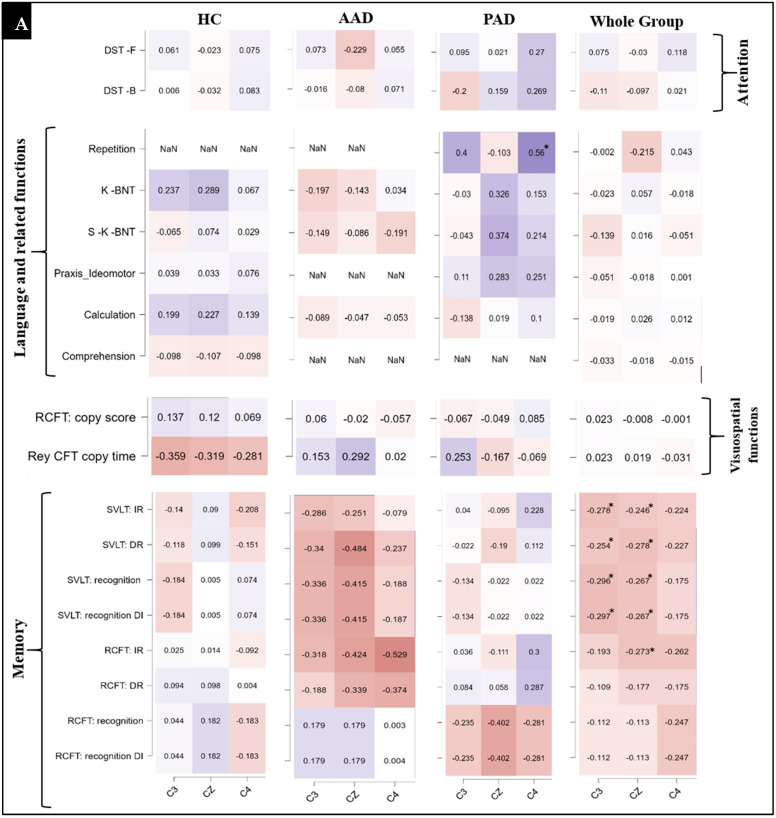

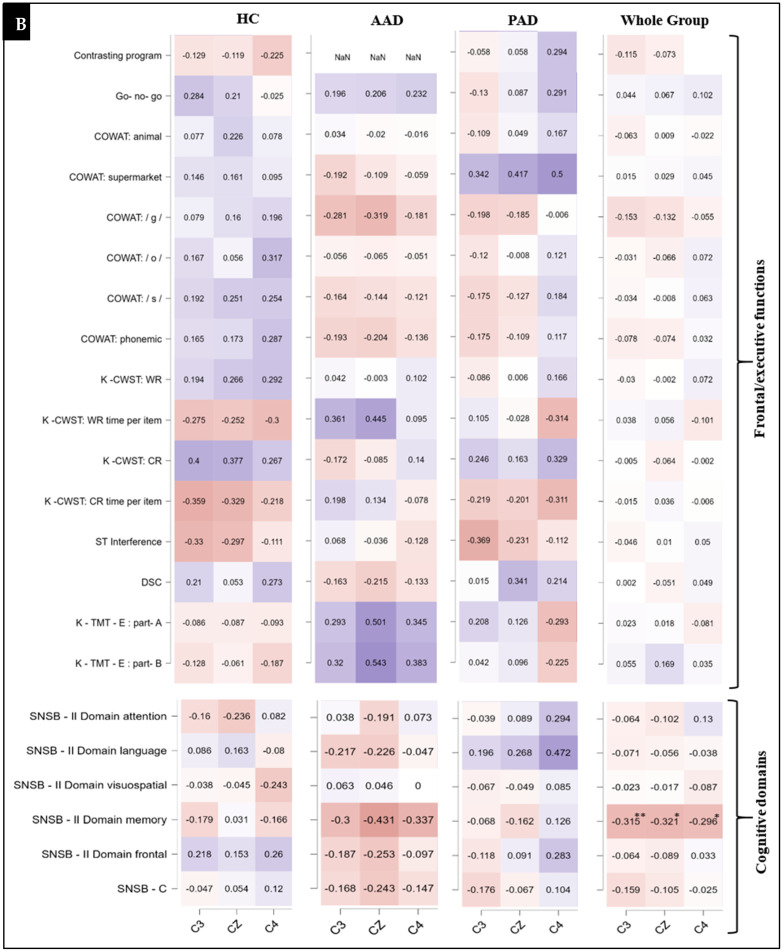
Correlations between the P300 peak latency and neuropsychological tests in the HC, AAD, and PAD groups and the whole-group correlation during the presentation of the target stimulus (**A**), and the correlation with frontal/executive tasks and among the cognitive domains (**B**). The level of significance is shown with (*)/* *p* < 0.05 and ** *p* < 0.01 (FDR-corrected).

Figure 6 summarizes the correlations between the neuropsychological test results and the P300 peak latency during the processing of the target stimulus. No significant correlations were observed between the neuropsychological tests and the P300 latency in the HC and AAD groups. However, in the PAD group, the Repetition test demonstrated a positive correlation with the P300 peak latency at the C4 electrode (r = 0.56, *p* = 0.035 *).

When performing the whole-group assessment, the memory domain primarily showed a significant negative correlation with the P300 peak latency at all of the three electrode sites, namely C3 (r = −0.315, *p* = 0.005 **), CZ (r = −0.321, *p* = 0.020 *), and C4 (r = −0.296, *p* = 0.04 *). In particular, there is a negative correlation between the P300 peak latency and the SVLT:IR and SVLT:DR subsets at the C3 (r = −0.278, *p* = 0.035 * and r = −0.254, *p* = 0.048 *) and CZ (r = −0.246, *p* = 0.046 * and r = −0.278, *p* = 0.034 *) electrodes. In addition, there is a negative correlation between the P300 peak latency and the SVLT:recognition subset at the C3 (r = −0.296, *p* = 0.032 *) and CZ (r = −0.267, *p* = 0.034 *) electrodes and the SVLT:recognition DI subset at the same electrodes (C3: r = 0.297, *p* = 0.032 * and CZ: r = −0.267, *p* = 0.034 *). For the RCFT set, our assessment showed a negative correlation between the P300 peak latency and the RCFT:IR subset at the CZ electrode (r = −0.273, *p* = 0.034 *).

## 4. Discussion

In this study, we investigated the potential use of P300 as a biomarker for cognitive dysfunction in the early stages of Alzheimer’s disease (AD), specifically asymptomatic AD (AAD) and prodromal AD (PAD). Previous research has examined the use of P300 indices, such as peak amplitude and latency, derived from oddball tasks, as potential biomarkers for the distinction between HC and MCI (PAD) [43,44,45,46]. Moreover, some studies have examined these indices in conjunction with neuropsychological tests [36,37,47,48,49,50,51,52,53]; however, to our knowledge, no previous studies have addressed the changes in P300 indices for patients with AAD.

While many studies have focused on identifying the neuroimaging, neuropsychological, or biological biomarkers associated with AAD [5,6,54,55,56], none have specifically evaluated whether P300 indices can reliably differentiate AAD from HC and PAD, either alone or in combination with neuropsychological assessments. Furthermore, the results remain inconsistent when using P300 indices, especially peak amplitude, with or without neuropsychological tests, to distinguish HC from MCI (PAD). As a result, our study aims to address this gap while providing more clarity rectifying the existing inconsistencies.

We begin by addressing the use of the P300 peak amplitude as a potential biomarker for differentiating AAD from HC and PAD. Our study revealed no significant differences in the P300 peak amplitude between the AAD and HC groups or between the AAD and PAD groups. Since the P300 amplitude reflects the allocation of attentional resources during cognitively demanding tasks [53], and because it has been reported that other domains except attention are impaired in the early stages of AD [57], it is understandable that a significant difference was not observed between the HC and AAD groups in terms of the P300 amplitude index. The insignificant difference found when comparing AAD to PAD is possibly due to the fact that the cognitive abilities of the AAD group are similar to normal people [58,59,60]; in addition, many studies have reported no significant difference in terms of the P300 amplitude when comparing MCI (PAD) and HC groups [31,32,33]. Therefore, we can conclude that the attention abilities of AAD subjects remains intact and that the P300 amplitude cannot serve as a reliable biomarker for distinguishing AAD from HC and PAD.

To address the lack of consistency among the different studies aiming to determine whether the P300 amplitude varies between those with PAD and HCs, we investigated the group differences using all 32 channel electrodes. Our findings indicated no significant differences in the P300 peak amplitude. This lack of difference could be attributed to the simplicity of the task [61], as several studies [23,37,43,45,46,48,51,53] have reported no group differences while employing oddball tasks. Such tasks may not be sufficiently complex to address the cognitive processes underlying the generation of P300. The limited complexity of these tasks might restrict our ability to detect subtle neural deficits in PAD. Furthermore, it is noteworthy that studies utilizing more complex executive functioning tasks [62,63] have demonstrated greater sensitivity to group differences, indicating that the design of the task plays a critical role in revealing the neurophysiological distinctions between these populations. Consequently, we can conclude that the P300 amplitude observed in the oddball task cannot serve as a reliable biomarker for distinguishing the PAD group from HCs.

After obtaining these results, we agreed upon the appropriateness of using P300 latency rather than its amplitude as a biomarker. A recent study [64] demonstrated that P300 latencies are more significant than amplitudes, and may be used either alone or in combination with amplitudes for the assessment of cognitive function. Therefore, we shifted our focus to latency as a potentially more relevant marker.

Regarding P300 latency, our study found no significant difference between the AAD and HC groups. This lack of difference may be attributed to the fact that AAD is associated with early pathological brain changes, such as amyloid-β accumulation, that have not yet manifested as clinical symptoms, including cognitive decline [65,66]. It is worth mentioning that our findings revealed a peak latency value that was later by 20 ms in the AAD group compared to the HC group at the CZ electrode, with most electrodes showing later latencies and minor electrodes showing similar latency values. This could serve as an indicator of the memory dysfunction associated with AAD, aligning with the results of [6,67], which reported the presence of accelerated memory decline in AAD using neuroimaging and neuropsychological biomarkers. Additionally, [51] demonstrates that the changes in P300 become more pronounced in the clinical stages of the disease, reinforcing the likelihood that no significant differences will be observed in the preclinical phase of AD. This suggests that the P300 peak latency alone may not be sensitive enough to detect subtle neurophysiological alterations at this early stage.

On the contrary, a significant difference in P300 latency was observed between the AAD and PAD groups, and between the HC and PAD groups, with the PAD groups exhibiting a prolonged P300 latency compared to the HC group, which is consistent with previous research [36,37,46,47,48,49,50,51,52], and the AAD group, which we believe is attributable to the higher working memory demands observed in PAD.

In general, the P300 latency reflects the processing of post-stimulus information, including individuals’ classification speed and executive functions [67,68,69] such as attention and memory. Based on this, our results indicate slower cognitive processing in individuals with PAD when identifying and classifying target stimuli. This demonstrates that the executive functions and memory tasks involved in stimulus processing are impaired in individuals with PAD.

From the aforementioned results, we can conclude that the P300 peak latency alone can serve as a biomarker for distinguishing individuals with AAD from HCs and those with PAD; however, it does not appear to be effective in differentiating those with AAD from HCs. This finding prompted us to explore the correlation between P300 latency (from electrodes that show the highest discrimination) and neuropsychological test results, as this could significantly enhance the early identification and characterization of neural dysfunction and thus provide opportunities for earlier differential diagnosis [61] and effective discrimination between groups.

In order to determine which electrodes would be able to discriminate between these groups most accurately, we reduced the number of electrodes to 12, and these electrodes were placed over the frontal, central, parietal, and occipital regions. By applying ANOVA analysis, we concluded that the central region is the best region for investigating the correlation, as it reflects the most significant difference (*p* < 0.001). This conclusion is consistent with the results of [17,70,71]. Accordingly, we selected the C3, CZ, and C4 electrodes as representatives of the central region, as their ANOVA test yielded a *p*-value less than 0.001 among the three groups.

Prior to investigating the correlation between the P300 peak latency and neuropsychological tests, we conducted an ANOVA analysis for these tests (attention, language and related functions, visuospatial functions, memory, and frontal executive functions) across the three groups. Our findings revealed no significant differences across tasks between the AAD and HC groups, accounting for the importance of investigating the aforementioned correlations. Remarkably, the PAD group exhibited a worse performance in the repetition language task, SVLT memory task, and SNSB-C compared to the AAD group, and a worse performance in the SVLT memory task compared to the HC group.

When investigating the correlation between the P300 peak latency and neuropsychological tests across all groups, we observed promising results. There was a negative correlation between the P300 peak latency and almost all memory function tasks (SVLT: IR, DR, recognition, recognition DI, and RCFT: IR). Moreover, a strong negative correlation was identified between the SNSB-II memory domain and the P300 peak latency at the C3, CZ, and C4 electrodes. This suggests that the P300 peak latency at these electrode sites, along with memory-related neuropsychological tasks, can serve as a biomarker for differentiating those with AAD from HCs and those with PAD. From a practical point of view, using only the memory-related neuropsychological assessment is more efficient as it shortens the overall duration of the experiment and alleviates the burden on senior subjects.

Some limitations should be acknowledged when interpreting the present results. First, the sample size included in our study is relatively small, and the population from which the sample was derived lacks demographic and cultural diversity. These limitations are common and challenging in most ERP studies, and we plan to include larger and more diverse cohorts in future studies to improve the statistical power and generalizability of our findings. Second, we rely on a single cognitive task, i.e., the visual oddball task, to conclude our findings. While it is a well-established protocol for eliciting P300 responses, it does not capture the full spectrum of cognitive deficits present in preclinical AD. Future research could integrate additional cognitive tasks to provide a more comprehensive assessment. Moreover, combining P300 latency measurements with multimodal imaging techniques, such as functional near-infrared spectroscopy (fNIRS), could enhance the diagnostic utility of this biomarker. Finally, we used the correlation between the P300 peak latency and memory-related neuropsychological tests to differentiate between the early stages of AD. While this approach demonstrates promise, its practical implementation in clinical settings requires further validation to ensure its reliability and reproducibility, particularly among diverse populations and experimental setups.

## 5. Conclusions

Asymptomatic Alzheimer’s disease (AAD) and prodromal Alzheimer’s disease (PAD) are transitional stages between normal cognitive aging and Alzheimer’s disease dementia. Research indicates that individuals with AAD and PAD are at a higher risk of developing dementia, highlighting the importance of identifying this disease early in order to implement timely intervention and treatment strategies. In this study, we explored the potential use of the P300 peak latency as a biomarker for distinguishing AAD from PAD and healthy controls (HCs). Our findings demonstrated that while the P300 peak latency effectively differentiates AAD from PAD, it is less reliable in distinguishing those with AAD from HCs. This limitation emphasizes the need for more precise methods, prompting us to investigate the correlation between the P300 peak latency and neuropsychological assessments conducted according to the Seoul neuropsychological screening battery. Our results revealed a strong negative correlation between the P300 peak latency and memory-related tasks, suggesting that combining memory-related tasks with the P300 peak latency could serve as a reliable biomarker for differentiating those with AAD from HCs and those with PAD. Moreover, from a practical perspective, focusing exclusively on memory-related assessments would streamline the experimental process, reduce time requirements and minimize the burden on older participants.

## Figures and Tables

**Table 1:** Demographics, psychometric, and statistical comparisons among groups.

	HC	AAD	PAD	*p*-Value
Count	35	22	22	-
Age (years)	74.8 ± 4.7	76.5 ± 5.1	79.6 ± 4.8	0.002
Education	10.4 ± 4.6	11 ± 3.8	11.6 ± 4.5	0.589
Gender (M/F)	9/26	15/7	15/7	<0.001 *
CDR	0.20 ± 0.25	0.13 ± 0.23	0.37 ± 0.23	0.009
MMSE	27.7 ± 1.8	27.9 ± 1.8	26.6 ± 2.1	0.066
K-MMSE	27.5 ± 1.9	27.8 ± 1.7	26.6 ± 1.9	0.067

CDR, Clinical Dementia Rating; MMSE, Mini-Mental State Examination; K-MMSE, Korean-Mini-Mental State Examination. * Chi-square test.

**Table 2:** Performance of groups on the neuropsychological battery and the results of the statistical analysis.

	HC	AAD	PAD	*p*-Value	HC vs. AAD	HC vs. PAD	AAD vs. PAD
**Attention**							
DST-F	5.7 ± 1.4	6.2 ± 1.1	6 ± 1.1	0.435	0.603	1.00	1.00
DST-B	3.7 ± 1	3.5 ± 0.9	3.3 ± 0.9	0.254	1.00	0.316	1.00
**Language and related functions**							
Repetition	15 ± 0	15 ± 0	14.8 ± 0.5	**0.007**	1.00	**0.011**	**0.025**
K-BNT	49.1 ± 6.8	50.3 ± 6.2	46.6 ± 7.9	0.217	1.00	0.619	0.266
S-K-BNT	12.5 ± 1.5	12.8 ± 1.5	12 ± 2.1	0.248	1.00	0.846	0.296
Praxis Ideomotor	4.9 ± 0.2	5 ± 0	4.8 ± 0.6	0.092	1.00	0.255	0.116
Calculation	10.8 ± 2.1	11.2 ± 1.3	10.3 ± 2.6	0.314	1.00	1.00	0.389
Comprehension	5 ± 0.2	5 ± 0	5 ± 0	0.539	1.00	1.00	1.00
**Visuospatial functions**							
RCFT: copy score	34.1 ± 2.3	33.8 ± 1.8	33.6 ± 3	0.753	1.00	1.00	1.00
RCFT: copy time	209.3 ± 93.9	241.9 ± 144.5	252.4 ± 127.3	0.364	0.955	0.565	1.00
**Memory**							
SVLT: IR	20.9 ± 3.7	20.7 ± 5.1	17.2 ± 4	**0.004**	1.00	**0.004**	**0.021**
SVLT: DR	6.6 ± 2.1	6.4 ± 2.8	4.9 ± 2.8	**0.042**	1.00	**0.046**	0.174
SVLT: recognition	21.1 ± 1.5	20.5 ± 2.5	19.2 ± 3.3	**0.018**	1.00	**0.014**	0.213
SVLT: recognition DI	88 ± 6.5	85.6 ± 10.3	80.1 ± 13.6	**0.018**	1.00	**0.014**	0.213
RCFT: IR	16.4 ± 7.6	17.7 ± 7.2	11.1 ± 7	**0.007**	1.00	**0.028**	**0.011**
RCFT: DR	16.2 ± 7	16.7 ± 6.5	11.6 ± 6.6	**0.023**	1.00	**0.044**	**0.044**
RCFT: recognition	19.9 ± 1.6	20 ± 1.7	18.9 ± 2.2	0.079	1.00	0.134	0.147
RCFT: recognition DI	83 ± 6.8	83.3 ± 6.9	78.8 ± 9.1	0.079	1.00	0.134	0.147
**Frontal/executive functions**							
Contrasting program	19.7 ± 1.2	20 ± 0	19.5 ± 1.9	0.433	1.00	1.00	0.611
Go-no-go	19.7 ± 1	19 ± 3.6	19 ± 2.8	0.504	1.00	0.904	1.00
COWAT: animal	15.5 ± 4.5	16.2 ± 3.5	14 ± 4.2	0.176	1.00	0.503	0.213
COWAT: supermarket	16.9 ± 4.8	17.2 ± 5.4	15.1 ± 5.2	0.314	1.00	0.580	0.508
COWAT:/g/	9.3 ± 3.7	11.3 ± 4	8.2 ± 3.5	**0.028**	0.177	0.872	**0.026**
COWAT:/o/	9.2 ± 4.2	10.5 ± 3.9	8.1 ± 4.4	0.163	0.742	1.00	0.174
COWAT:/s/	9.9 ± 4.2	10.2 ± 3.8	8.9 ± 4.6	0.560	1.00	1.00	0.909
COWAT: phonemic	28.4 ± 10.9	32 ± 10.2	25.3 ± 11.6	0.127	0.672	0.888	0.130
K-CWST: WR	111.6 ± 11.4	111.6 ± 0.7	111 ± 2.5	0.264	1.00	0.405	0.515
K-CWST: WR time per item	0.6 ± 0.1	0.6 ± 0.1	0.7 ± 0.2	0.363	1.00	0.695	0.579
K-CWST: CR	92.2 ± 16.2	89.5 ± 17.5	76.4 ± 12.8	**0.001**	1.00	**0.001**	**0.021**
K-CWST: CR time per item	1.3 ± 0.3	1.4 ± 0.3	1.6 ± 0.3	0.018	1.00	0.016	0.153
ST Interference	0.7 ± 0.2	0.7 ± 0.3	0.9 ± 0.2	**0.022**	0.931	**0.017**	0.309
DSC	49.6 ± 18.8	49.4 ± 15.5	43.3 ± 15.1	0.353	1.00	0.542	0.712
K-TMT-E: part-A	30.9 ± 23.2	24.8 ± 8.1	28.7 ± 20.3	0.510	0.743	1.00	1.00
K-TMT-E: part-B	52.1 ± 33.2	51.1 ± 30.5	57.7 ± 21.1	0.734	1.00	1.00	1.00
**SNSB-II total**							
SNSB-II domain attention	9 ± 1.6	9.7 ± 1.7	9.3 ± 1.9	0.381	0.498	1.00	1.00
SNSB-II domain language	0.199 ± 0.2	0.248 ± 0.2	0.042 ± 0.4	**0.031**	1.00	0.103	**0.038**
SNSB-II domain visuospatial	0.469 ± 0.5	0.532 ± 0.3	0.5 ± 0.4	0.851	1.00	1.00	1.00
SNSB-II domain memory	0.238 ± 0.6	0.232 ± 0.7	−0.374 ± 0.7	**0.002**	1.00	**0.004**	**0.011**
SNSB-II domain frontal	0.241 ± 06	0.361 ± 0.5	−0.009 ± 0.5	0.082	1.00	0.304	0.089
SNSB-C	23.4 ± 7.1	27.4 ± 6.1	22.2 ± 6.6	**0.028**	0.096	1.00	**0.035**

HC, healthy control; AAD, asymptomatic AD; PAD, prodromal AD; DST: Digit Span Test, F: forward, B: backward; K-BNT: Korean-Boston Naming Test; S-K-BNT: Short Form of the K-BNT; RCFT: Rey Complex Figure Test; SVLT: Seoul Verbal Learning Test, IR: immediate recall, DR: delayed recall, DI: discriminability Index; COWAT, Controlled Oral Word Association Test; K-CWST, Korean-Color Word Stroop Test, WR: word reading, CR: color reading; ST, Stroop Test; DSC, Digit Symbol Coding, K-TMT-E, Korean-Trail Making Test; SNSB-II, Seoul Neuropsychological Screening Battery, 2nd Edition; SNSB-C, Seoul Neuropsychological Screening Battery—Core.

## Data Availability

The datasets used and/or analyzed during the current study are available from the corresponding author upon reasonable request.
